# Kyasanur Forest Disease and Alkhurma Hemorrhagic Fever Virus—Two Neglected Zoonotic Pathogens

**DOI:** 10.3390/microorganisms8091406

**Published:** 2020-09-12

**Authors:** Bharti Bhatia, Heinz Feldmann, Andrea Marzi

**Affiliations:** Laboratory of Virology, Division of Intramural Research, National Institute of Allergy and Infectious Diseases, National Institutes of Health, Hamilton, MT 59840, USA; bharti.bhatia@nih.gov (B.B.); feldmannh@niaid.nih.gov (H.F.)

**Keywords:** KFDV, AHFV, vector distribution, human disease, animal models, pathogenesis, countermeasures

## Abstract

Kyasanur Forest disease virus (KFDV) and Alkhurma hemorrhagic fever virus (AHFV) are tick-borne flaviviruses that cause life-threatening hemorrhagic fever in humans with case fatality rates of 3–5% for KFDV and 1–20% for AHFV, respectively. Both viruses are biosafety level 4 pathogens due to the severity of disease they cause and the lack of effective countermeasures. KFDV was discovered in India and is restricted to parts of the Indian subcontinent, whereas AHFV has been found in Saudi Arabia and Egypt. In recent years, both viruses have spread beyond their original endemic zones and the potential of AHFV to spread through ticks on migratory birds is a public health concern. While there is a vaccine with limited efficacy for KFDV used in India, there is no vaccine for AHFV nor are there any therapeutic concepts to combat infections with these viruses. In this review, we summarize the current knowledge about pathogenesis, vector distribution, virus spread, and infection control. We aim to bring attention to the potential public health threats posed by KFDV and AHFV and highlight the urgent need for the development of effective countermeasures.

## 1. Introduction

Kyasanur Forest disease virus (KFDV) and Alkhurma hemorrhagic fever virus (AHFV) belong to the tick-borne encephalitis virus serocomplex of flaviviruses that cause severe hemorrhagic fever in humans [1,2,3]. KFDV was first identified in 1957 in sick and dying monkeys from the Kyasanur Forest of Karnataka, India [4,5]. Since then, annually 400–500 human cases have been recorded with a case fatality rate of ~3 to 5% [6,7]. AHFV was first isolated in Jeddah, Saudi Arabia, in the 1990s from blood samples of butchers reported to have a hemorrhagic disease [8]. The case fatality rate for AHFV in humans is 1–20% [9]. AHFV shares a remarkable nucleotide sequence homology of 89% with KFDV and, therefore, has been considered a variant of KFDV [2,10,11]. It has been hypothesized that AHFV might have arisen from an introduction of KFDV to Saudi Arabia [12]. Both viruses are classified as category C priority pathogens by the National Institute of Allergy and Infectious Diseases and handled exclusively in Biosafety level 4 (BSL4) laboratories due to their life-threatening pathogenicity and the absence of effective vaccines or treatments. 

Being zoonotic and vector-borne in origin, KFDV and AHFV are circulated between a tick vector and a vertebrate host. *Haemaphysalis spinigera* and *Ornithodoros savignyi* are the main tick vectors involved in KFDV and AHFV transmission, respectively [13,14]. *H. spinigera* is distributed in India, Sri Lanka, and Vietnam. About 95% of KFDV isolates have been obtained from this tick species. Ticks take up a vertebrate blood meal during different development stages acquiring the virus that is subsequently transmitted to the host. Transstadial transmission of the virus has also been reported [15]. Seasonal outbreaks of human KFDV and AHFV occur during spring and summer which correlates with the maximum activity of the respective tick vector [16,17,18]. KFDV has been isolated from rodents, birds, cattle, and bats; however, only primates seem to develop disease [16,19]. Humans contract KFDV infections through bites of infected ticks or by handling infected mammalian and avian species. No human-to-human transmission has been reported for KFDV so far (Table 1). AHFV has been epidemiologically linked with camel and sheep [17]. Humans become infected with AHFV either by bites of infected ticks or contact with infected blood or milk [20]. Similar to KFDV, human-to-human transmission for AHFV is not known. Interestingly, sequence analysis indicated that AHFV diverged from KFDV approximately 700 years ago [12]. Analysis of the envelope gene E of AHFV revealed the circulation of three sub-lineages (I–III) suggesting a continuous evolution [11]. Recently, AHFV emerged in Egypt demonstrating a range expansion to a larger area than previously known [21,22].

KFDV and AHFV are neglected human pathogenic viruses, and there remains a lack of insight into their pathogenesis, ecology, and epidemiology and the development of effective countermeasures. In this review, we summarize the current knowledge and emphasize the urgent need for more basic and applied research and countermeasure development to combat these significant public health threats.

## 2. Virus Biology

**Figure 1:**
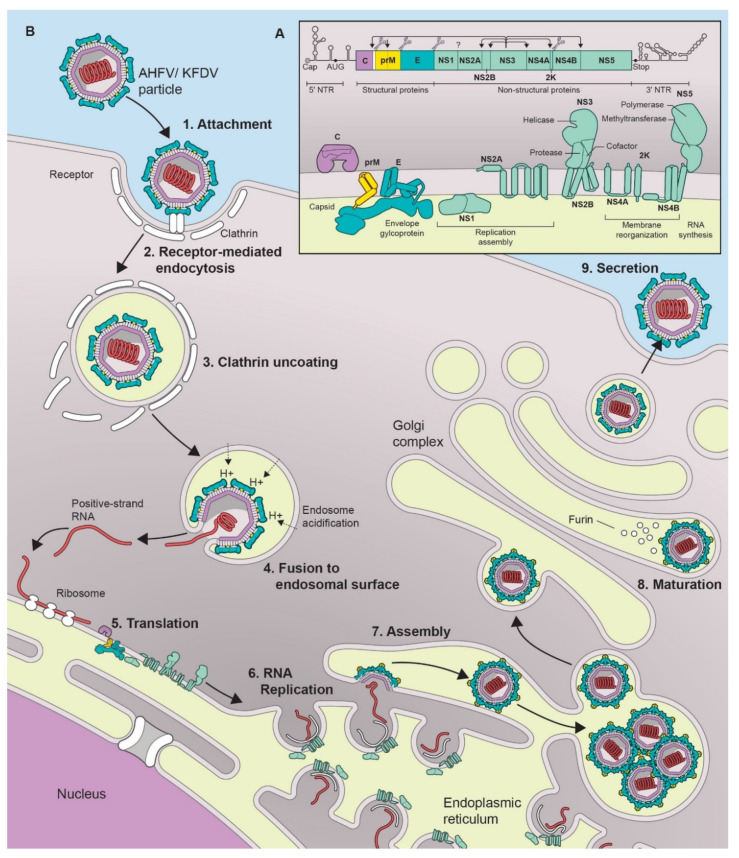
Flavivirus replication cycle and genome structure. (**A**) Schematic of a flavivirus genome, polyprotein, and the mature viral proteins. (**B**) Lifecycle of a typical flavivirus.

KFDV and AHFV belong to the genus *Flavivirus* in the family Flaviviridae [3]. As with all flaviviruses, the virus particles are 40–65 nm in size with an icosahedral nucleocapsid [12]. The genome consists of a positive sense single-stranded RNA which is 10,774 nucleotides (nt) in length and encodes a single polyprotein that is post-translationally cleaved into a total of 3 structural proteins (capsid (C), precursor membrane protein (prM), and envelope protein (E)), and 7 non-structural (NS) proteins (NS1, NS2A, NS2B, NS3, NS4A, NS4B, and NS5) [3,10,12] (Figure 1A).

Flaviviruses share a typical virus replication cycle which is also the case for KFDV and AHFV (Figure 1B). The envelope protein (E) binds to the cell surface using attachment factors such as glycosaminoglycans, and subsequently the virus enters the cell by receptor-mediated, clathrin-dependent endocytosis [23]. Several cell surface receptor candidates involved in flavivirus entry have been described [24] including α_v_β_3_ integrins [25], C-type lectin receptors (CLR) [26,27,28,29], laminin receptor [30], and phosphatidylserine receptors [31]. The endocytic vesicles containing the virion further traffic to endosomes, where the acidic environment triggers the dissociation of the dimeric form of the E protein into monomers and further reassociation into trimers. This conformational change leads to particle disassembly, fusion of the viral and endocytic membrane and release of the viral genome into the cytoplasm [32,33]. The positive-stranded RNA gets translated into a single precursor polypeptide at ribosomes bound to the surface of the endoplasmic reticulum (ER). The polypeptide is then cleaved by viral and host proteases into the individual proteins [34]. Genome replication occurs on ER-derived host cell membranes induced by the virus that act as a platform for the attachment of the viral replication complexes and host cell factors required for replication [35]. NS1, in its dimeric form, is required for formation of the replication complex and recruitment of other non-structural proteins to the ER-derived membrane structures. It is also secreted as a hexameric lipoparticle that plays a role against host immune evasion by antagonizing the complement function and inhibiting signal transduction originating from Toll-like receptor 3 [36]. NS2A and NS2B are part of the viral replication complex [37]. NS3 in association with NS2B, acts as a serine protease that auto-cleaves the polyprotein at dibasic sites in the cytoplasm. NS3 also acts as a NTPase and RNA helicase that binds to RNA and unwinds dsRNA in the 3’ to 5’ direction. NS4A regulates the ATPase activity of NS3 during RNA unwinding. NS4B induces the formation of ER-derived membrane vesicles for virus replication and inhibits interferon (IFN)-induced STAT1 phosphorylation to maintain the cellular antiviral state [38]. NS5 is an RNA-dependent RNA polymerase, that replicates the viral genome, but also possesses methyl- and guanylyl transferase activity required for the genome capping in the cytoplasm [39]. It also helps maintain the cellular antiviral state by preventing activation of the JAK-STAT signaling pathway [40]. 

The viral RNA structure also plays a role in translation and replication [41,42]. The 5′ untranslated region (UTR) is composed of two elements, the large stem loop (SLA), ∼70 nt long domain at the extreme 5′ terminus, and the small stem loop (SLB) which is present downstream of SLA [43]. The SLA element is a Y-shaped structure that is recognized by the viral RNA polymerase (NS5) [44]. The SLB element contains the AUG initiation codon. At the 3′end of SLB lies a highly conserved hairpin that governs the selection of the translation initiation codon by directly positioning the ribosomal complex close to the “functional” AUG in the SLB element. The 3′UTR is about 700 nt long. It lacks a poly(A) tail; instead it ends in a conserved CU_OH_ dinucleotide [44]. The 3′UTR is subdivided into three regions, domains I-III. Domain I is located downstream of the translation stop codon. It is a hypervariable sequence followed by two conserved stem loop (SL) domains. The secondary structures formed by these SL domains play a role as regulatory replication elements [45]. Domain II has a secondary structure consisting of three helices, also known as a dumbbell. These elements play a role in maintaining efficient translation. Domain III is composed of terminal genomic functional elements, the short hairpin (sHP) and the 3′SL. The sHP acts as a potential recruitment site for protein factors involved in the establishment of RNA–RNA interactions, and the 3’SL element plays a role in in viral replication by facilitating the formation of a “replication complex”.

Finally, the viral RNA is complexed with the C protein [46] and the virion particle is packaged into an ER-derived lipid bilayer containing hetero-dimers of the prM and E proteins [47]. The C protein aids in virus budding by binding to the ER membrane and packaging the viral genome [46]. The virions are then transferred to the Golgi complex for the final maturation process during which furin-mediated cleavage converts prM into M [32,35]. This process ensures that the premature virion does not fuse with the cell membrane during the export process. Mature infectious particles are released into the extracellular medium by Golgi-derived exocytosis.

## 3. Epidemiology

**Figure 2:**
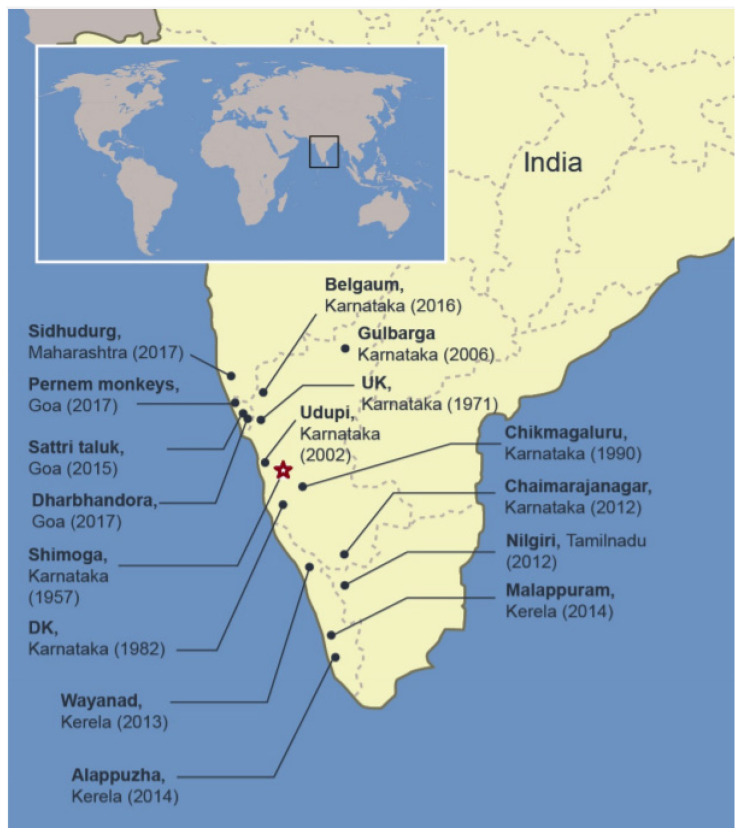
KFD distribution in India. Areas in the south western part of India where KFD in humans and monkeys has been reported are indicated. Place, state, and year in which the first case was reported are provided. Star represents the place of the first isolation from a monkey. UK Uttar Kannada, DK Dakshina Kannada.

After the discovery of KFDV in 1957 in the Kyasanur Forest, Shimoga District of Karnataka, a southern state of India, outbreaks have occurred almost annually in this district (Figure 2). High mortality of monkeys, black-faced langurs (*Semnopithecus entellus)* and red-faced bonnet macaques (*Macaca Radiata),* was generally observed from December to May, during the active period of nymphal *Haemaphysalis* ticks [48]. Human encroachment in the affected area heightens the encounters with infected ticks. Human activity peaks post monsoon for the paddy harvest, gathering firewood and other forest products [49]. Annual outbreaks had case numbers ranging from 200–500 [50]. The cases were restricted to the Shimoga district, Karnataka. In 1971, new cases were reported in the Uttar Kannada (UK) district of Karnataka and in 1982, cases were reported from Dakshina Kannada. In the following years, the disease spread to other Karnataka districts, namely Chikmagalur and Udupi. Then in 2001, over 400 cases were reported in Shimoga and UK, Karnataka. In 2002, the number of cases increased to over 600. The growth in the number of cases continued in 2003 reaching over 900 cases. Subsequently, new cases emerged from other districts of Karanataka namely Gulbarga, Chamarajanagar and Belgaun. A total of 3263 human cases were reported from Karnataka between 2003 and 2012 with 823 confirmed cases and 28 confirmed deaths [6]. In recent years, an expansion of KFDV’s endemic area is worrisome as the cases no longer remain restricted to the Karnataka state. KFDV infection of monkeys has been confirmed from the Nilgiri district of the Tamil Nadu State and from Kerala State, the neighboring states of Karnataka [51]; the first human case in this area was confirmed in the Wayanad district, Kerala, in May 2013 [52]. In 2014, cases were also confirmed from the Malappuram and Alappuzha districts of Kerela. There was an outbreak in Wayanad in 2015 causing numerous fatal monkey infections and 18 confirmed human cases. In 2015, human KFD cases were also reported from the Sattari taluk area in the state of Goa including nine deaths [51]. KFDV emerged again in Parnem and Dharbhandora taluks from the same state. Meanwhile, KFDV presence was confirmed from the Sindhudurg district, Maharashtra State in 2016 [53,54]. These outbreaks confirm the expansion of the endemic area of KFDV (Figure 2).

**Figure 3:**
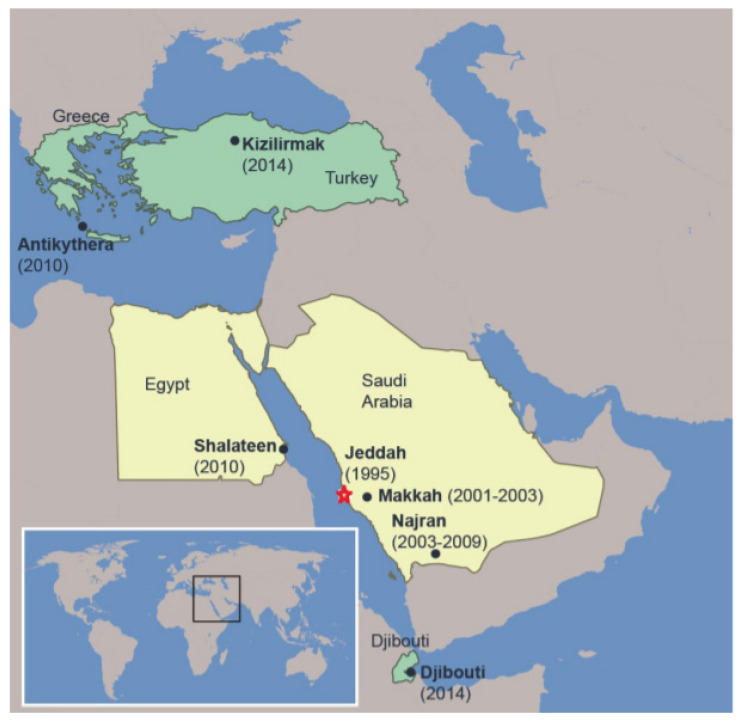
AHFV distribution in middle eastern countries. Yellow areas indicate human disease caused by AHFV in Egypt and Saudi Arabia. Greece, Turkey, and Djibouti (depicted in green) represent the places were viral RNA was found in ticks. Place, state, and year in which the first case was reported are provided. Star represents the place of the first human case.

AHFV was first described with the isolation of the virus from a butcher in Jeddah, Saudi Arabia in 1995 (Figure 3) [54]. Additional cases were reported in Makkah from 2001–03, and in Najran from 2003–09, in the south of Saudi Arabia [17,55]. Interestingly, soldiers in the eastern and northern provinces of Saudi Arabia have been found seropositive for AHFV [56]. Later, in 2010, two Italian travelers returning from Shalateen, Egypt, were reported to be infected with AHFV [21,22]. Further reports also suggested the presence of the disease in Djibouti, at the Horn of Africa, where AHFV RNA was isolated from ticks, and a single native in Djibouti was found to be seropositive for AHFV [20,57]. AHFV RNA was also detected in immature *Hyalomma rufipes* ticks infesting northward migratory birds like *Motacilla flava*, *Lanius senator niloticus* and *Acrocephalus schoenobaenus,* which were caught in Antikythera, Greece (2010), and Kizilimark, Turkey (2014) (Figure 3) [58].

Amid rising cases of KFDV and AHFV infections, there has been tremendous progress in both the diagnosis and reporting of new cases [51,59]. The initial symptoms of both diseases are the same as with other viral diseases like Dengue, Rift valley fever (RVF) and Crimean–Congo hemorrhagic fever (CCHF), which also have overlapping endemic areas. This fact might have resulted in misdiagnosis and under-reporting of the actual cases for AHF and KFD in the past. The first patient reported to contract AHFV was originally suspected to have CCHFV [8]. Retrospective reports suggest that it is possible that KFDV may have been present in India before 1957, but cases may not have been identified or reported [1,48]. Before 1957, the incidents of increased mortality in local NHPs in India was assumed to be caused by plague or influenza virus rather than a new disease [60]. However, a single human KFDV case has also been reported from China, in a region close to the Indian border [61] indicating that there might be more. 

## 4. Geographical Aspects Contributing to Virus Spread

The evergreen forests of the Western Ghats provide the ideal topographical and climate conditions for tick vectors; thus, making these areas an epitome for tick-borne diseases. The rapid spread of KFDV has been largely attributed to human encroachment of the forest for poaching, collection of firewood and cashews as well as clearance of the forest for agricultural and industrial land needs [62,63,64]. First, it renders higher risk of direct exposure of humans to the infected ticks. Second, changes in the landscape encourage the migration of host species to new forest habitats, thereby changing the population dynamics of ticks as well. Black-faced langurs, the natural hosts of KFDV, are being hunted for meat by local communities. Because of the poaching activity, the animals are under a higher pressure for migrating to a new area, thereby, increasing the possibility of disease transmission [65]. Moreover, highly mobile hosts such as bats and birds play a potential role in spreading the disease as they can disperse ticks to wider areas [66,67]. Two frugivorous bats species, namely *Rousettus leschenaulti* and *Cynopterus sphinx*, have been demonstrated to circulate experimentally inoculated KFDV supporting this hypothesis [68]. Cleared forest areas tend to get covered with shrubs offering a favorable environment for rodents and birds which subsequently act as hosts for the growing tick larvae and nymphs [48]. Third, deforestation further affects the local pattern of precipitation, thus impacting the micro-climate of the region. The arthropod distribution and lifecycle heavily depend on the temperature and precipitation of a region. The tick larval population gets activated during the dry season [69]. Temperature also directly influences the transmission efficiency of the vector to a vertebrate host [70,71]. It has been estimated that the area of the dense forests of the Western Ghats has been reduced by about 19% over the last two decades impacting the regional climate [72]. 

More research is required to understand the geographical and demographical factors underlying AHFV spread. The emergence of tick-borne diseases in non-endemic areas may be associated with pathogen dissemination caused by the movements of livestock and migratory birds [73,74]. Over a million sheep, cows, and camels are transported and slaughtered during the annual pilgrimage in Makkah and Mina [75,76,77], putting the pilgrims from countries worldwide at risk. According to records of the Food and Agriculture Organization, Somalia at the Horn of Africa exported a record 5.3 million animals in 2014 [78]. Recently, AHFV RNA was detected in immature *Hyalomma rufipes* ticks infesting northward migratory birds caught in the North Mediterranean Basin [58]. Another factor that might affect the distribution of the AHFV tick vector, is climate change [71]. There has been significant increase in the annual temperature of Saudi Arabia over the last few decades [79,80]. Higher temperatures directly influence the transmission efficiency of a vector to a vertebrate host [70,71] and result in closer proximity of the vectors to animal housing and potential exposure to humans [81].

## 5. Ecology

Both KFD and AHF are zoonotic diseases. In nature, these viruses are transmitted between their tick vector and multiple mammalian hosts; humans serve as a dead-end host for both viruses. 

KFDV has been isolated from various genera of ticks such as the *Haemophysalis, Ixodes* [1,14,82], *Argas, Ornithodoros, Hyalomma*, *Dermacentor*, and *Rhipicephalus* [63]. However, *H. spinigera* is believed to be the main vector as 95% of the existing KFDV isolates originate from this species [52,83,84]. In the tick vector, KFDV is transovarially and transstadially transmitted and a tick, once infected, remains infected throughout its entire life cycle. Ticks have stage-specific activity levels. Humans get infected through the bite of nymphs and adult ticks which are mostly active during the dry season in India. The larval population builds up during the monsoon months and becomes active when the litter dries up during the post monsoon months [16,52,85]. Larvae feed on smaller animals and rarely transmit the virus to larger animals or humans. However, nymphs and adults are more agile, feed for days as they need a lot of blood for molting. Their activity is high from January to May each year, overlapping with the occurrence of most of the human KFD cases during the year [16]. 

KFDV has a wide range of hosts including humans, rodents (forest rats, shrews, white-bellied rat, white-tailed rat, and squirrels), bats, ground dwelling birds, Indian crested porcupines, and monkeys (black-faced langur, bonnet macaque, and gray langur) [86]. The red-faced bonnet macaques and black-faced langurs have been described to be highly susceptible to KFDV infection [87]. Low levels of viremia have been detected in rats, bats, and cattle [16,68,88]. KFDV antibodies were found in goats, shrews, birds, and other species [6,87]. Cows shed the virus in their urine, dispersing it in the environment [16]. They also harbor masses of adult ticks favoring co-feeding of ticks, which potentially enables viral transmission between ticks without infection of the host [89]. Bats have been shown to be parasitized by infected ticks and KFDV has been isolated from the insectivorous bat *Rhinolophus rouxi* [68]. When humans visit areas with sick and dying monkeys such as woodlands and forests their chances of getting exposed to the infected ticks increase [52]. While tick-to-human transmission is an established route of infection, direct human-to-human transmission has not yet been reported.

Much less is known about the ecology of AHFV. *Ornithodoros savignyi* and *Hyalomma dromedarii* are the known vectors of AHFV [13,90]. Recently, AHFV RNA was also detected in immature *Hyalomma rufipes* ticks infesting northward migratory birds caught in the North Mediterranean Basin [58]. No animal reservoirs have been reported for AHFV. Livestock animals, mainly camels and sheep, are epidemiologically linked to AHFV. These animals are ectoparasitized by infected ticks; thus, they only act as hosts for transmission between ticks as well as tick amplification [91]. Evidence points towards more than one possible route of transmission. Humans can get infected via tick bite or contact with infectious blood through a wound [18]. Like KFDV, human-to-human transmission of AHFV has not yet been documented. 

## 6. Human Disease

KFDV and AHFV cause hemorrhagic fever in humans with an incubation period of 2–4 days. The symptoms begin with high fever of about 40 °C, headache, body aches in the neck, upper, and lower back and extremities, diarrhea, anorexia, insomnia, vomiting, myalgia, cough, photophobia due to conjunctival inflammation of the sclera, and hemorrhagic manifestations such as bleeding from gums, nose, or the gastrointestinal tract resulting in hemoptysis and melena [4,6,54,85,86,92,93,94]. Patients exhibit signs such as decreased heart rate and blood pressure, display abnormal blood chemistry with elevated liver enzymes, elevated creatinine phosphokinase and elevated blood urea nitrogen levels [8,95], have altered hematology such as reduction in eosinophils, neutrophils, and lymphocytes within the first week of illness with the neutrophil count dropping below 2000 cells/mL [92,96]. Between the third and the fifth week after onset of disease, lymphocytosis has been observed in several patients. In some cases, radiographic findings show pneumonitis. After the onset of illness, the amount of virus circulating in the blood is high, therefore, is easily detected in and isolated from human serum samples [5,6,61,97]. Currently, the human infectious dose for KFDV and AHFV is unknown. Histopathological findings encompass hepatomegaly with parenchymatic degeneration, nephrosis, along with increased erythrophagocytosis in the spleen [96]. Patient samples from the acute febrile but not from the convalescent phase showed elevated levels of circulating type I IFN [98].

Some KFDV patients (10–20%) develop a secondary phase of fever relapse [86] with neurological manifestations including mental disturbance, drowsiness, transient disorientation, confusion, convulsion, tremors and loss of consciousness [99,100]. AHFV patients do not display this secondary phase of fever relapse; however, about 10% of AHFV patients display primary neurological signs including seizures, encephalitis, and severe muscular weakness [13,22,54]. Patients recovering from KFDV sometimes continue to have hand tremors or unsteadiness for several weeks which eventually resolve [1,4,101]. Long-term sequelae are rare.

## 7. Diagnosis

The rising number of KFDV and AHFV cases combined with the fact that human infections occur in an overlapping geographical area with other prevalent diseases such as CCHF and RVF, which cause similar clinical symptoms [9] demand faster and reliable methods for detection. Historically, diagnosis was done by virus neutralization test [102], hemagglutination inhibition test [103], complement fixation test, and inoculation of patient specimens into mice [85]. Not only are these techniques time consuming, they are not appropriate for early detection of an infection. More recent techniques rely on detection of the virus directly from patient blood as the virus titer rises rapidly after infection. 

The first line of testing for KFDV and AHFV infections is RT-PCR and RT-qPCR, respectively [7,10,91]. These tests rely on the amplification of the NS5 region of the viral genome from a patient’s blood sample. Humans display high titer viremia (about 10^6^ pfu /mL) around day 3 post onset of symptoms for up to two weeks [5,6]. As these tests are quick, very specific, and highly sensitive, a virus load as low as 10 copies/mL can be detected [7,10]. 

Another diagnostic method, the enzyme-linked immunosorbent assay (ELISA), is used to detect KFDV/AHFV-specific antibodies in human serum plasma or serum [104,105]. IgM antibodies can be detected around five days after symptom onset for about three months. IgG antibodies can be detected around five days after symptom onset for about four months [104]. ELISA-based detection is especially important when determining the serostatus of a population in a particular area. For example, KFDV-specific antibodies were found in the population of many states of India such as Gujarat and Maharashtra, West Bengal, and Andaman and Nicobar Islands [105].

## 8. Animal Models

Most animal infection studies with KFDV were performed in the 1960s. As KFDV was first found in tissue homogenates derived from a dead monkey [4,5], KFDV pathogenesis was initially studied in non-human primate (NHP) models. In nature, bonnet macaques and black-faced langurs can fall victims to KFDV infection [87,106]. However, macaques are more extensively studied because langurs are endangered, and their size makes them difficult to be used in laboratory settings. Bonnet macaques represent a severe and often lethal model for KFDV [107]. The disease manifestation is similar to humans; however, the lethality rate is approximately 85%. Macaques infected with KFDV show hematological changes such as thrombocytopenia and leucopenia, develop elevated alkaline phosphatase (ALP) and alanine transaminase (ALT) enzyme levels as well as phagocytosis of nuclear material in the peripheral blood and reticulo–endothelial system [108]. The disease is largely hemorrhagic with occasionally accompanying encephalitis. KFDV is distributed systemically, and necrosis has been observed in liver, spleen, kidney and occasionally the gastrointestinal tract. The stomach and intestines undergo cryptic loss as well as fusion of villi. Comparison of the disease progression in macaques and langurs demonstrated that KFDV infection in both the species lead to high-titered viremia and death. Hence, these studies provide valuable models for understanding KFDV pathogenesis [5,109].

Comparative studies of KFDV and AHFV in immunocompetent mice demonstrated that AHFV infection leads to replication in visceral tissues, induces pro-inflammatory cytokine responses and hematological abnormalities [110,111]. KFDV however, was found to be lethal in both young (3–4-week-old) and adult (≥5 weeks old) mice infected by the subcutaneous, intraperitoneal and intranasal route [112]. The disease progression and pathology are very different from that of NHPs or humans. High viral titers are detected mostly in the brain, but not in other tissues [110,111]. Occasionally, the virus replicated to low levels in the lungs [111]. Hence, in mice, KFD displays as a neurological rather than a hemorrhagic disease. Mice succumb to disease between 7- and 10-days post infection. Enlarged spleen and limited evidence of hemorrhage in the liver were also observed accompanied by serum abnormalities like an increase in liver transaminases and hypoalbuminemia [93,96,110,111]. Studies have shown that most of the mice that survive a KFDV infection do not generate a detectable level of neutralizing antibody titers and succumb to disease when re-infected with KFDV [113]. Therefore, the utility of the mouse model for KFDV lies more in countermeasure development than pathogenesis. KFDV also infects other mammalian animal species, such as rat, gerbil, porcupines, shrews, squirrels, and cattle [19]. Infections are asymptomatic leading to KFDV-specific antibody responses. For AHFV, other than the comparative mouse studies [110,111], no other animal species have been evaluated. 

## 9. Prevention and Treatment

The first vaccine against KFDV was generated by the Indian Council of Medical Research (ICMR) based on a formalin-inactivated mouse-brain formulation of Russian spring–summer encephalitis virus (RSSEV) due to its genetic and antigenic similarities to KFDV [114]. Unfortunately, the immune responses elicited by the RSSEV vaccine were insufficient to combat and prevent KFDV [115,116]. Later, an inactivated KFDV vaccine was prepared by growing the virus in newborn Swiss albino mice brains followed by formalin inactivation [117]. The vaccine induced neutralizing antibodies in mice but had a very short shelf life. Another KFDV vaccine approach was followed by propagating the virus in chick embryos; unfortunately, this vaccine was only weakly immunogenic and failed to induce protective immune responses in mice [118,119]. Efforts were also made to generate a live-attenuated vaccine by weakening the pathogenicity of the KFDV P9605 strain through serial passaging in cell culture [120]. The vaccine failed to provide protection against challenge in langurs. In 1966, a formalin-inactivated vaccine was generated by propagating KFDV in chick embryo fibroblast cultures. This vaccine is immunogenic, protective, safe and stable and still used today [121,122]. However, a study conducted by Kasabi et al., showed that the efficacy of this vaccine in humans was only about 62% when two doses were administered, and 83% with multiple booster vaccinations [123]. Currently, the strategy of human KFDV vaccination is following a prime and boost approach a month apart followed by additional booster vaccinations [123]. However, vaccine coverage is low in endemic areas and vaccine storage remains a problem. At present, there is no vaccine available against AHFV.

Currently, no anti-viral drugs are available to treat KFDV- or AHFV-infected patients. At present, patient management includes maintenance of proper hydration and circulation by transfusion of intravenous fluids, colloids and electrolytes, maintaining oxygen status and blood pressure, and treatment for any medical complications [124] depending on clinical signs and symptoms. 

Since both KFDV and AHFV are tick-borne diseases, emphasis is given to preventive measures with focus on avoiding tick contact in their respective areas of risk. Tick repellants are distributed among people living in endemic areas and insecticides are sprayed in the affected regions. Pamphlets are distributed to raise awareness about the disease. People are advised to avoid tick-infested areas, use tick repellants on skin and check themselves for attached ticks as soon as possible after contact. Tick collars are also available for domestic animals and the use of acaricides has been shown to be effective in killing ticks on livestock [17,125,126,127].

## 10. Future Directions

Short-term efforts should be put into increasing the awareness of KFD and AHF. When entering forested areas in endemic locations, the use of insect repellant, body-covering clothing and tick removal should be practiced. People should be encouraged to seek early diagnosis upon tick manifestation and first clinical symptoms. Additionally, people should be encouraged to report viewings of dead monkeys as an early warning sign of risk of infection. New diagnostic assays need to be developed and healthcare infrastructure needs to be improved to allow for proper deployment in endemic areas. Since KFDV and AHFV infections often occur in remote locations, simple, rapid, and field deployable assays would be the first choice. Non-invasive clinical specimen collection would be preferable as diagnostic samples.

Mid-term efforts should be put into the development of surveillance programs. As KFD and AHF are zoonotic diseases occurring in remote areas, interdisciplinary approaches to understand vector and reservoir ecology are needed. Surveillance programs should be focusing on relevant arthropod, avian and mammalian species involved in the life and transmission cycle of the pathogens. Tick surveillance should inform about distribution and expansion due to climate changes allowing to define larger at risk regions for exposure to KFDV and AHFV. Recently, birds and bats became of interest in their role of spreading infected ticks during seasonal migration. Accordingly, radio tracking of certain migratory bird species should be included in such programs. Overall, detailed modeling of various parameters influencing the geographical distribution of the disease would provide insight into the circumstances surrounding virus and disease emergence. 

Long-term efforts should be focused on countermeasure development. Currently, the only KFDV vaccine available is only partially protective in humans [128] and vaccine coverage is limited [123]. New vaccines based on different platforms should be developed, keeping in mind the remote and often inaccessible endemic areas with challenges for logistics of vaccine administration. For better public health control of KFD and AHF, target group vaccination or, during outbreaks, areal vaccination might be preferred approaches. In addition to the classical vaccine approaches, the development of a universal anti-tick vaccine (vaccine against tick saliva proteins) should be considered. For treatment, better knowledge of virus biology, host-pathogen interaction and pathogenesis would be helpful in identifying promising drug targets. Drug screening systems should be developed allowing the identification of repurposed and new drugs for further preclinical and clinical development. 

## 11. Conclusions

KFDV and AHFV are BSL4 pathogens and select agents in the United States of America and represent an increasing threat to human and animal health as endemic areas are expanding. Many factors have been described to influence the distribution of both viruses as well as their epidemiology including deforestation, migration of vectors and reservoir hosts, increased human contact with infected animals, and climate change. With an extended distribution of the tick vectors, greater areas are at risk. In addition, it appears that migratory birds and bats may play an underestimated role in the dissemination of KFDV and AHFV presenting an even higher potential threat to public health. Therefore, more attention should be given to these neglected emerging tick-borne pathogens. Intensifying studies on ecology and epidemiology will help to build surveillance systems allowing to track emergence and spread. Better understanding of virus biology and pathogenesis will help to define targets for countermeasure development. Vaccination seems the preferred prophylactic measure likely using population targeted and areal vaccination approaches. Not less important are advanced diagnostics and therapeutic intervention for patient management and outbreak control. 

## Figures and Tables

**Table 1:** Characteristics of Kyasanur Forest disease virus (KFDV) and Alkhurma hemorrhagic fever virus (AHFV).

	KFDV	AHFV
**Geographic distribution**	India	Saudi Arabia and Egypt
**First outbreak**	1957	1995
**Tick vector**	*Haemophysalis spinigera*	*Ornithodoros savignyi*
**Mode of transmission**	Tick bite, encounter with dead or dying monkeys	Tick bite, contact with animal fluids such as milk or blood
**Natural hosts**	Black-faced langur, Red-faced bonnet macaque, rodents, shrews, birds	Unknown
**Animal models**	Bonnet macaques, mice	Mice
**Vaccine**	Formalin-inactivated virus	None
**Human-to-human transmission**	Unknown	Unknown
**Case fatality rate**	3–5%	1–20%
